# COVID-19 associated CKM syndrome progression in diabetic patients is linked to pancreatic beta cell dysfunction, rather than RASi use: a retrospective cohort study

**DOI:** 10.3389/fendo.2026.1790281

**Published:** 2026-03-09

**Authors:** Yongcheng Zhang, Ziqi Wang, Yizhe Wang, Ran Bao, Liping Gu, Na Li, Yuhang Ma, Xiaoying Ding

**Affiliations:** 1 School of Health Science and Engineering,University of Shanghai for Science and Technology, Shanghai, China; 2 Department of Endocrinology and Metabolism, Shanghai General Hospital, Shanghai Jiao Tong University School of Medicine, Shanghai, China; 3 School of Medicine, University of Electronic Science and Technology of China, Chengdu, China

**Keywords:** CKM syndrome, COVID-19, diabetes mellitus, propensity score matching, RASI, β-cell dysfunction

## Abstract

**Background and aims:**

SARS-CoV-2 primarily has tissue tropism for the respiratory epithelium; however, it may cause multiple organ dysfunction due to the widespread expression of its entry receptor, ACE2. Specifically, SARS-CoV-2 may enter pancreatic β-cells by exploiting ACE2, disrupting cellular and hormonal activity, and thereby worsening Cardiovascular-Kidney-Metabolic (CKM) syndrome prognosis in diabetic patients. Furthermore, treatment with renin-angiotensin system inhibitors (RASi) may arguably increase ACE2 expression and facilitate viral entry, making its independent impact highly debated. Accordingly, we sought to elucidate the genuine impact of RASi usage on CKM progression via Propensity Score Matching (PSM) and concurrently investigate the specific contribution of pancreatic β-cell secretory dysfunction.

**Methods:**

We conducted a retrospective investigation involving 682 diabetic patients across CKM stages 2 to 4 with confirmed SARS-CoV-2 infection utilizing multivariate logistic regression coupled with 1:1 PSM to rectify selection bias concerning RASi administration and identifying predictors.

**Results:**

Post-infection CKM progression was observed in 25.2% of the cohort and while initial multivariate models implicated RASi (OR = 1.56, 95% CI: 1.06–2.28; p=0.02) as a risk factor this association lost statistical significance after rigorously balancing baseline characteristics through PSM (OR = 1.56, 95% CI: 0.91–2.67; p = 0.156). A paradoxical finding emerged wherein the progression group exhibited low HOMA-IR scores accompanied by reduced fasting C-peptide and elevated glucose thereby indicating severe viral-induced β-cell secretory dysfunction rather than enhanced insulin sensitivity while High-Density Lipoprotein Cholesterol (HDL-C) persisted as a protective factor.

**Conclusions:**

COVID-19 exacerbates CKM progression in diabetic patients, but this is not driven by RASi therapy itself. RASi use should be interpreted as a “marker of severity” for baseline comorbidities, rather than a pathogenic factor. Mechanistically, viral infection may cause the failure of compensatory mechanisms in pancreatic β-cell function (manifested as low C-peptide and spurious low HOMA-IR).

## 1 Introduction

Cardiovascular-kidney-metabolic (CKM) syndrome is a multisystem disorder characterized by the interplay of metabolic risk factors, chronic kidney disease (CKD), and cardiovascular disease (CVD). Its pathophysiology centers on a vicious cycle involving insulin resistance, chronic inflammation, and organ dysfunction (1). Within the progression of CKM syndrome, type 2 diabetes mellitus (T2DM) serves not only as a key driver but also as a central nexus linking multiorgan damage. Hyperglycemic toxicity, adipokine dysregulation, and dyslipidemia directly impair vascular endothelium and glomerular basement membranes while accelerating atherosclerosis and renal interstitial fibrosis via oxidative stress pathways (2, 3). Substantial clinical data corroborates the capacity of diabetes to intensify CKM syndrome pathophysiology thereby resulting in markedly elevated heart failure-associated morbidity and mortality among diabetic cohorts relative to non-diabetic controls and concurrently driving a 2.76-fold escalation in the hazard of deteriorating toward dialysis necessity specifically within the context of coexisting CKD (4, 5).

This is further exacerbated by SARS-CoV-2 infection, which causes COVID-19 and may increase insulin resistance because of cytokine storms in the body, in addition to directly affecting pancreatic β-cells via ACE2 receptor-mediated dedifferentiation or apoptosis and subsequent acute failure in insulin secretion (6, 7). This virus-induced hormonal imbalance leads to unregulated Hyperglycemia, thus accelerating the progress of CKM syndrome (8, 9). Collectively, ample scientific data supports an intensified risk profile for CVD progression, rapid deterioration of renal functions, and unrelenting glucometabolic derangements in diabetic patients post-COVID-19; however, the meager assessment of risk factors within this particular subgroup hinders the design of patient-tailored therapeutic plans (10–15).

Renin-angiotensin system inhibitors (RASi) represent core therapeutic cornerstones in high-risk populations of CKM; nevertheless, there has also been an emergence of controversy bordering upon the relative safety of these compounds in the setting of the COVID-19 pandemic owing to conflicting speculations regarding an exaggerated role of “upregulation of ACE2” facilitating viral entry into target cells versus proven anti-inflammatory and cardiorenal-protective effects (16, 17). While some non-controlled studies have suggested an association of adverse outcome with administered use of RASi, these findings have also had to contend with “confounding by indication,” suggesting that patients expressing conditions such as hypertension or proteinuria tend to be preferentially selected for these agents (18). How to exclude the interference of the severity of underlying diseases and assess the true impact of RASi on CKM progression remains an urgent clinical problem to be addressed.

This study aims to systematically evaluate the key drivers of CKM syndrome progression in diabetic patients following COVID-19 infection. We specifically employ PSM to rigorously adjust for baseline confounding factors, thereby re-evaluating the true association between RASi use and CKM progression. Additionally, we integrate fasting C-peptide levels with HOMA indices to explore in depth the potential role of pancreatic β-cell function status in disease progression, so as to provide a basis for risk stratification and precise intervention in high-risk CKM populations.

## 2 Materials and methods

### 2.1 Participants

A retrospective single-center analysis was conducted on a cohort of 698 diabetic patients with verified COVID-19 infection recruited from the Metabolic Management Center at Shanghai General Hospital in China. The research protocol obtained formal approval from the Medical Ethics Committee of the aforementioned institution (2017KY209-5C24-1) while written informed consent was secured from all participants in full compliance with the ethical standards mandated by the Declaration of Helsinki.

**Figure 1:**
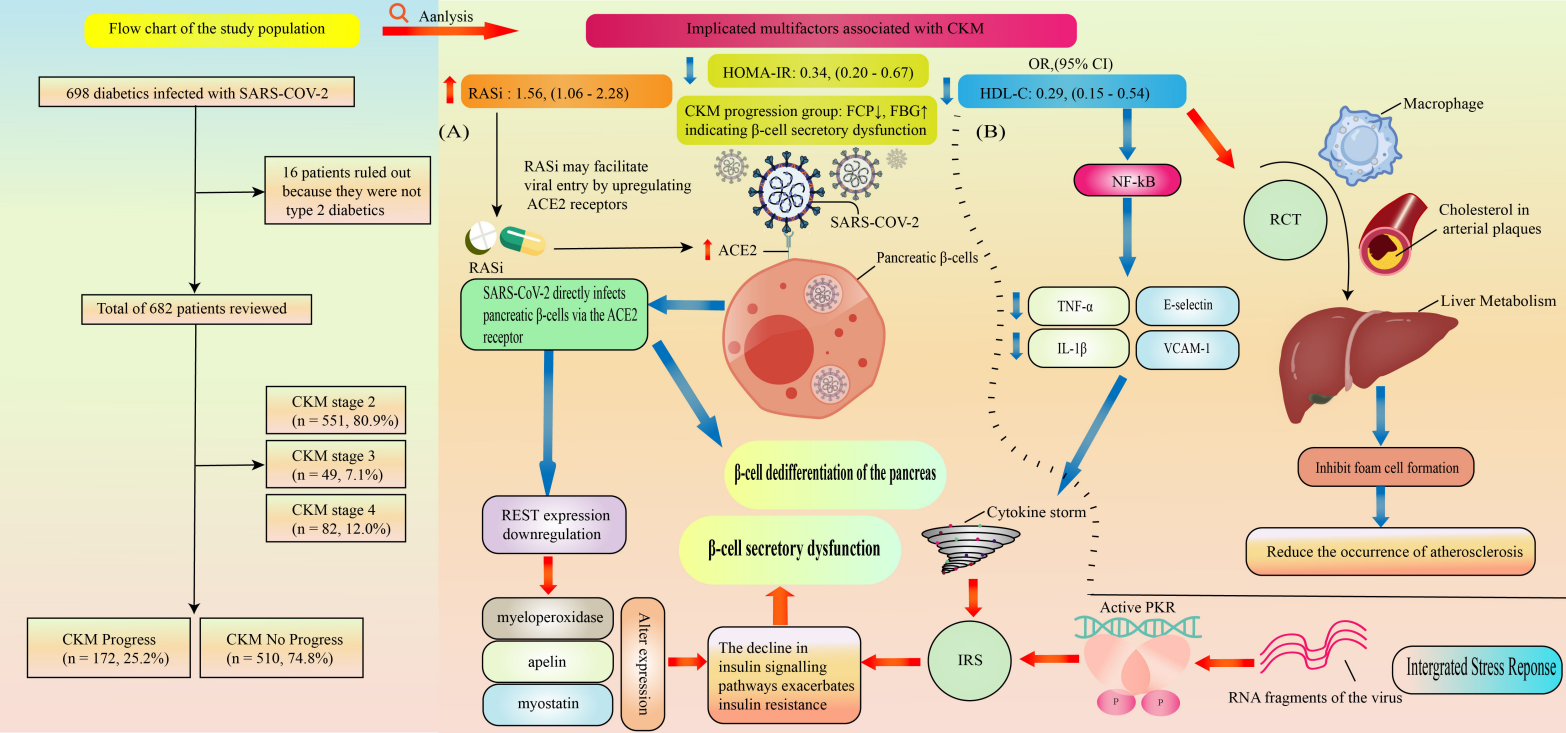
Flow chart of the study population and Graphical Abstract. **(A)** Viral-Induced β-Cell Injury: SARS-CoV-2 directly invades pancreatic β-cells via the ACE2 receptor, triggering two distinct pathological pathways: (1) Dedifferentiation: The virus forces β-cells to lose their insulin-secreting phenotype; and (2) Molecular Stress and Functional Failure: Viral infection downregulates the transcription factor REST and activates the Integrated Stress Response (ISR). These molecular alterations exacerbate intracellular insulin resistance and induce endoplasmic reticulum (ER) stress, ultimately leading to irreversible β-cell secretory failure. **(B)** Pleiotropic Protective Effects of HDL-C: (1) Reverse Cholesterol Transport (RCT): HDL-C promotes cholesterol efflux from macrophages in atherosclerotic plaques to the liver for excretion, thereby inhibiting foam cell formation and attenuating atherogenesis. (2) Anti-inflammatory Action: HDL-C mitigates the COVID-19-associated “cytokine storm” by inhibiting NF-kB activation, which subsequently downregulates endothelial adhesion molecules (e.g., E-selectin, VCAM-1) and suppresses the release of pro-inflammatory cytokines (e.g., TNF-α, IL-1β).

The investigative timeline was designed to span both pre-pandemic and pandemic intervals wherein 698 diabetic patients underwent baseline evaluations antecedent to infection followed by post-infection assessments conducted between July 2021 and April 2023 which uniformly comprised comprehensive protocols involving detailed questionnaires and anthropometrics alongside standardized steamed-bun meal tests and fasting blood sampling. Subsequently the exclusion of sixteen non-type 2 diabetic individuals yielded a definitive cohort of 682 patients stratified by baseline CKM severity into Stage 2 (n=551, 80.9%) and Stage 3 (n=49, 7.1%) as well as Stage 4 (n=82, 12.0%) as illustrated in **Figure 1**. The electronic health records collected comprehensive data, including but not limited to demographic data, clinical parameters, glycometabolic indicators, liver function parameters, lipid profile parameters, renal function parameters, lifestyle, disease history, and medication history data.

### 2.2 Definition of CKM conditions

CKM staging was operationalized based on the guidelines issued by the American Heart Association President which classify the syndrome into progressive tiers: Stage 0 indicates a risk-free status; Stage 1 involves excess adiposity or adipose dysfunction; and Stage 2 signifies the coexistence of metabolic determinants like diabetes and hypertension with CKD (1). Subsequently Stage 3 is marked by subclinical cardiovascular pathology as inferred from very high-risk CKD per KDIGO standards or elevated 10-year CVD risk via the AHA PREVENT model while Stage 4 comprises patients with documented clinical cardiovascular events including peripheral arterial disease or stroke regardless of renal failure status (19, 20).

Renal impairment was defined according to the KDIGO, glomerular filtration rate was calculated according to the blood creatinine-based CKD-EPI equation developed by the U.S. Chronic Kidney Disease Epidemiology Collaboration Working Group in 2009, and participants were categorized into four CKD risk classes: Low risk(GFR = G1 or G2 and Albuminuria = A1)、Moderately increased risk(GFR = G1 or G2 and Albuminuria = A2, GFR = G3a and Albuminuria = A1)、High risk(GFR = G1 or G2 and Albuminuria = A3, GFR = G3a and Albuminuria = A2, GFR = G3b and Albuminuria = A1)、Very high risk(GFR = G3a and Albuminuria = A3, GFR = G3b and Albuminuria = A2 or A3, GFR = G4 or G5 and Albuminuria = A1 or A2 or A3) (19, 21). Moderately increased risk, High risk and Very high risk are recognized as CKD (9, 22).

The diagnostic criteria for metabolic syndrome (MetS) are defined as the presence of metabolic syndrome diagnosed by at least three of the following five indicators: (1) BMI ≥ 23 kg/m² in an Asian population (2) systolic blood pressure ≥ 130 mmHg or diastolic blood pressure ≥ 80 mmHg and/or use of antihypertensive medications (3) TG ≥ 1.7 mmol/L (4) HDL-C < 1.0 mmol/L (men) or HDL-C < 1.3 mmol/L (women) (5) Fasting blood glucose ≥ 5.6 mmol/L or diagnosed type 2 diabetes mellitus (1, 23, 24). The CKM progression population was defined as patients with metabolic syndrome, CKD, and CVD added after COVID-19 infection.

### 2.3 Statistical analysis

Prioritizing data integrity variables exhibiting missingness beyond a 20% threshold were discarded while those falling below this limit underwent imputation via a Random Forest-based iterative regressor algorithm thereby ensuring dataset completeness.

Continuous metrics were represented as means with standard deviations for normal distributions or medians with interquartile ranges for skewed data whereas categorical variables were denoted by frequencies and percentages. Intergroup disparities were assessed utilizing one-way analysis of variance for normally distributed variables across CKM stages and unpaired Student’s t-tests for progression comparisons while non-parametric data required Kruskal-Wallis or Wilcoxon rank-sum tests and categorical variables were analyzed via Chi-square or Fisher’s exact tests.

To isolate independent predictors of CKM progression we executed multivariate stepwise logistic regression with bidirectional elimination quantifying associations via odds ratios with 95% confidence intervals. Crucially to mitigate confounding by indication regarding RASi usage we implemented PSM by constructing a multivariable logistic model to estimate scores based on baseline covariates and applying a 1:1 nearest-neighbor matching algorithm with a strict 0.05 standard deviation caliper whereby successful balancing was confirmed by non-significant differences in covariate distribution between matched groups. Statistical significance was determined by a two-sided P-value below 0.05 and all analytical procedures were facilitated using Python software version 3.12.7.

## 3 Results

Among the 682 diabetic patients included in the analysis, baseline CKM staging revealed the following distribution: 80.9% (n=551) in Stage 2, 7.1% (n=49) in Stage 3, and 12.0% (n=82) in Stage 4. Baseline characteristics stratified by CKM stage are presented in **Table 1**. Patients in Stages 3 and 4 were significantly older than those in Stage 2 (48.16 vs. 65.38 vs. 58.62 years, respectively; p < 0.01). Significant differences were observed in metabolic and therapeutic profiles across stages: patients in Stages 3 and 4 exhibited higher levels of HbA1c, glycated serum albumin, and creatinine (Cr), alongside increased usage of insulin, statins, and RASi compared to Stage 2 patients. Conversely, ALT, ALB, and eGFR were notably lower in advanced stages.

**Table 1:** Baseline characteristics of the study population stratified by CKM stages (Stages 2–4).

Characteristics^a^	Stage 2 (n = 551, 80.9%)	Stage 3 (n = 49, 7.1%)	Stage 4 (n = 82, 12.0%)	P
Sex				0.1
Male, n (%)	356,64.6%	39,79.6%	55,67.1%	
Female, n (%)	195,35.4%	10,20.4%	27,32.9%	
Age (years)	48.16 ± 11.32	65.38 ± 7.11	58.62 ± 10.26	<0.01
BMI (kg/m2)	25.10 ± 3.14	24.95 ± 2.71	24.54 ± 2.60	0.13
SBP (mmHg)	129.18 ± 14.45	139.04 ± 21.72	129.54 ± 16.29	<0.01
DBP (mmHg)	76.85 ± 9.06	75.53 ± 11.75	74.03 ± 7.88	0.03
HbA1C (%)	6.46 ± 0.85	6.91 ± 0.85	6.91 ± 1.00	0.04
Glycated Serum Albumin (%)	15.04 ± 3.22	16.34 ± 3.41	16.68 ± 3.34	<0.01
FBG (mmol/L)	6.33 ± 1.34	6.98 ± 1.56	6.59 ± 1.28	0.24
FCP (Pmol/L)	572.91 ± 292.40	571.27 ± 297.82	571.19 ± 297.13	0.65
HOMA-IR(CP)	2.98 ± 1.07	3.17 ± 1.17	3.14 ± 1.44	0.23
HOMA-β(CP)	68.96 ± 70.07	60.88 ± 66.72	61.06 ± 44.02	0.59
Hb (g/L)	151.47 ± 15.28	152.84 ± 11.45	146.55 ± 17.15	0.17
ALT (U/L)	25.89 ± 11.52	21.25 ± 8.51	23.96 ± 9.59	0.05
AST (U/L)	22.27 ± 6.20	19.2(16.63,23.40)	22.27 ± 6.37	0.36
ALP (U/L)	69.59 ± 17.29	73.91 ± 18.02	67.93 ± 18.40	0.57
γ-GT (U/L)	24.84 ± 13.73	22.2(15.05,32.45)	24.91 ± 14.32	0.73
ALB (g/L)	46.6 ± 2.98	45.18 ± 2.61	45.23 ± 3.41	<0.01
TG (mmol/L)	1.46 ± 0.66	1.76 ± 0.96	1.39 ± 0.65	<0.01
TC (mg/dl)	173.55 ± 39.70	191.16 ± 56.91	148.74 ± 40.84	<0.01
HDL-C (mmol/L)	1.06 ± 0.26	1.05 ± 0.33	1.09 ± 0.30	0.86
LDL-c (mmol/L)	2.63 ± 0.90	3.07 ± 1.36	2.13 ± 0.82	<0.01
TyG	422.05 ± 680.36	520.78 ± 765.40	586 ± 1186.59	0.36
BUN (mmol/L)	5.76 ± 1.36	6.38 ± 1.52	6.4 ± 1.62	0.12
eGFR (mL/min/1.73m²)	114.15 ± 20.11	98.79 ± 25.17	100.19 ± 20.21	<0.01
Cr (mg/dl)	0.73 ± 0.17	0.83 ± 0.21	0.81 ± 0.19	<0.01
UACR	15.19 ± 11.96	15.81(9.27,29.44)	12.22 ± 7.15	<0.01
UA (umol/L)	322.05 ± 80.68	323.35 ± 68.39	336.83 ± 79.08	0.25
Metformin, n (%)	348,63.2%	30,61.2%	51,62.2%	0.95
SGLT-2i, n (%)	382,69.3%	32,65.3%	62,75.6%	0.4
DPP-4i, n (%)	290,52.6%	31,63.3%	35,42.7%	0.06
Insulin, n (%)	98,17.7%	16,32.7%	26,31.7%	<0.01
Sulfonylureas, n (%)	9,1.6%	4,8.2%	1,1.2%	<0.01
GLP-1 RA, n (%)	185,33.6%	11,22.4%	33,40.2%	0.11
TZD, n (%)	22,4.0%	3,6.1%	2,2.4%	0.58
AGI, n (%)	149,27.0%	18,36.7%	29,35.4%	0.13
Statins, n (%)	311,56.4%	31,63.3%	69,84.1%	<0.01
RASi, n (%)	163,29.6%	27,55.1%	44,53.6%	<0.01
Hypertension, n (%)	179,32.5%	34,69.4%	55,67.1%	<0.01
Hyperlipidemia, n (%)	160,29.0%	17,34.7%	47,57.3%	<0.01
Hyperuricemia, n (%)	61,11.1%	2,4.1%	16,19.5%	0.02
Smoking, n (%)	145,26.3%	22,44.9%	15,18.3%	<0.01
Drinking, n (%)	174,31.5%	24,49.0%	24,29.3%	0.15

BMI, body mass index; SBP, systolic blood pressure; DBP, diastolic blood pressure; HbA1c, glycosylated Hemoglobin; FBG, fasting blood-glucose; FCP, fasting C-peptide; HOMA-IR(CP), homeostatic model assessment of insulin resistance(C-peptide); HOMA-β(CP), homeostatic model assessment of beta-cell function; Hb, hemoglobin(C-peptide); ALT, alanine aminotransferase; AST, aspartate aminotransferase; ALP, alkaline phosphatase; γ-GT, γ-glutamyl transpeptidase; ALB, albumin; TG, triglycerides; TC, total cholesterol; HDL-C, high-density lipoprotein cholesterol; LDL-C, low-density lipoprotein cholesterol; TyG, triglyceride-glucose; BUN, blood urea nitrogen; eGFR, estimated glomerular filtration rate; Cr, serum creatinine; UACR, morning urine albumin-to-creatinine ratio; UA, uric acid; SGLT-2i, sodium-dependent glucose transporters 2 Inhibitor; DPP-4i, Dipeptidyl Peptidase-4 Inhibitor; GLP-1 RA, glucagon-like peptide-1 receptor agonists; TZD, Thiazolidinedione; RASi, renin-angiotensin system inhibitors.

a Continuous variables were expressed as mean ± standard deviation for normally distributed data and as median (interquartile range, IQR) for skewed data. Categorical variables were reported as frequencies and percentages.

**Table 2** compares clinical profiles between patients with CKM progression (n=172, 25.2%) and those without progression (n=510, 74.8%). The progression group was older (mean age: 52.40 vs. 49.95 years, p = 0.03) and had a higher prevalence of pre-infection hypertension (44.2% vs. 30.9%, p < 0.01) and RASi usage (46.5% vs. 36.9%, p = 0.03).

**Table 2:** Comparison of general information at baseline between progressive and non-progressive DM in CKM.

Characteristics^a^	Progression (n = 172, 25.2%)	Non-progression (n = 510, 74.8%)	P
Sex			0.06
Male, n (%)	103,59.9%	347,68.2%	
Female, n (%)	69,40.1%	163,32.0%	
Age (years)	52.40 ± 12.67	49.95 ± 11.90	0.03
BMI (kg/m2)	25.59 ± 3.48	25.39 ± 3.85	0.53
SBP (mmHg)	129.16 ± 16.65	130.17 ± 16.62	0.49
DBP (mmHg)	75.06 ± 9.87	77.10 ± 10.32	0.02
HbA1C (%)	6.79 ± 1.25	7.05 ± 1.73	0.04
Glycated Serum Albumin (%)	15.60 ± 4.32	16.50 ± 5.43	0.03
FBG (mmol/L)	6.98 ± 1.56	6.33 ± 1.34	<0.01
FCP (Pmol/L)	552.49 ± 290.16	637.62 ± 410.51	<0.01
HOMA-IR(CP)	2.72 ± 0.70	3.11 ± 1.22	<0.01
HOMA-β(CP)	50.21(33.29,82.17)	49.97(31.34,84.53)	0.69
Hb (g/L)	149.80 ± 16.46	150.35 ± 16.62	0.71
ALT (U/L)	26.72 ± 13.89	31.87 ± 30.98	<0.01
AST (U/L)	22.56 ± 7.19	25.68 ± 19.31	<0.01
ALP (U/L)	70.87 ± 24.77	72.43 ± 22.66	0.47
γ-GT (U/L)	20.88(14.76,31.00)	23.05(15.45,37.55)	0.07
ALB (g/L)	46.13 ± 3.94	46.03 ± 3.64	0.76
TG (mmol/L)	1.63 ± 1.17	1.40(0.98,2.14)	0.28
TC (mg/dl)	163.83 ± 47.53	172.24 ± 53.37	0.05
HDL-C (mmol/L)	1.06 ± 0.27	1.11 ± 0.34	0.06
LDL-c (mmol/L)	2.57 ± 0.87	2.65 ± 1.03	0.36
TyG	206.99(136.76,352.12)	223.77(132.07,427.66)	0.36
BUN (mmol/L)	6.00 ± 1.66	5.80(4.90,6.80)	0.13
eGFR (mL/min/1.73m²)	110.93 ± 26.17	114.55 ± 25.82	0.12
Cr (mg/dl)	0.74 ± 0.24	0.78 ± 0.50	0.22
UACR	13.37(8.95,26.39)	12.66(7.40,27.26)	0.39
UA (umol/L)	327.11 ± 84.10	327.21 ± 84.69	0.99
Metformin, n (%)	112,65.1%	317,62.1%	0.55
SGLT-2i, n (%)	123,71.5%	353,69.2%	0.64
DPP-4i, n (%)	87,47.7%	269,52.7%	0.69
Insulin, n (%)	38,22.1%	102,20.0%	0.63
Sulfonylureas, n (%)	3,1.7%	11,2.2%	0.98
GLP-1 RA, n (%)	59,34.3%	170,33.3%	0.89
TZD, n (%)	4,2.3%	23,4.5%	0.3
AGI, n (%)	52,30.2%	144,28.2%	0.69
Statins, n (%)	111,64.5%	300,58.8%	0.21
RASi, n (%)	76,44.2%	158,30.9%	<0.01
Hypertension, n (%)	80,46.5%	188,36.9%	0.03
Hyperlipidemia, n (%)	59,34.3%	165,32.4%	0.7
Hyperuricemia, n (%)	22,12.8%	57,11.2%	0.66
Smoking, n (%)	46,26.7%	136,26.7%	0.99
Drinking, n (%)	50,29.1%	172,33.7%	0.31

BMI, body mass index; SBP, systolic blood pressure; DBP, diastolic blood pressure; HbA1c, glycosylated Hemoglobin; FBG, fasting blood-glucose; FCP, fasting C-peptide; HOMA-IR(CP), homeostatic model assessment of insulin resistance(C-peptide); HOMA-β(CP), homeostatic model assessment of beta-cell function(C-peptide); Hb, hemoglobin; ALT, alanine aminotransferase; AST, aspartate aminotransferase; ALP, alkaline phosphatase; γ-GT, γ-glutamyl transpeptidase; ALB, albumin; TG, triglycerides; TC, total cholesterol; HDL-C, high-density lipoprotein cholesterol; LDL-C, low-density lipoprotein cholesterol; TyG, triglyceride-glucose; BUN, blood urea nitrogen; eGFR, estimated glomerular filtration rate; Cr, serum creatinine; UACR, morning urine albumin-to-creatinine ratio; UA, uric acid; SGLT-2i, sodium-dependent glucose transporters 2 Inhibitor; DPP-4i, Dipeptidyl Peptidase-4 Inhibitor; GLP-1 RA, glucagon-like peptide-1 receptor agonists; TZD, Thiazolidinedione; RASi, renin-angiotensin system inhibitors.

a Continuous variables were expressed as mean ± standard deviation for normally distributed data and as median (interquartile range, IQR) for skewed data. Categorical variables were reported as frequencies and percentages.

Notably, although the progression group showed lower HOMA-IR (2.72 vs. 3.11, p < 0.01), this was accompanied by significantly reduced fasting C-peptide levels (p < 0.01) and higher fasting glucose levels (6.98 vs. 6.33 mmol/L, p < 0.01). This paradoxical “low HOMA-IR, low C-peptide” profile suggests severe SARS-CoV-2-induced β-cell secretory dysfunction rather than improved insulin sensitivity.

The results of the univariate and multivariate logistic regression analyses are presented in **Table 3**. In Model 1, it appeared that the use of ACE inhibitors/ARBs was associated with increased risk (OR = 1.56, p=0.02). However, after the addition of hypertension and other variables into Model 2, this association became non-significant (OR = 1.30, 95% CI: 0.85-1.99, p=0.23). This indicates that the association may have been due to the “confounding by indication.”

**Table 3:** Univariate and multivariate logistic regression analysis of risk factors for CKM progression in the original cohort (pre-matching).

Characteristics	Univariate		Multivariate model 1		Multivariate model 2	
	Odds ratio (95% CI)	P	Odds ratio (95% CI)	P	Odds ratio (95% CI)	P
Sex (Male)	0.70(0.49,1.00)	0.04	0.61(0.41,0.90)	0.01	0.50(0.31,0.79)	0.01
Age (years)	1.02(1.00,1.03)	0.02	1.01(1.00,1.03)	0.08	1.01(0.99,1.01)	0.12
BMI (kg/m2)	1.02(0.97,1.06)	0.52			1.06(1.00,1.13)	0.03
SBP (mmHg)	0.98(0.96,1.00)	0.03				
HbA1c (%)	0.90(0.90,1.03)	0.12				
FBG (mmol/L)	0.84(0.75,0.92)	<0.01				
FCP (Pmol/L)	1.00(1.00,1.00)	0.03	1.00(1.00,1.00)	<0.01		
HOMA-IR(CP)	0.68(0.55,0.83)	<0.01	0.34(0.20,0.67)	<0.01	0.58(0.45,0.74)	<0.01
ALT (U/L)	0.99(0.98,1.00)	0.03				
AST (U/L)	0.98(0.96,1.00)	0.03				
HDL-C (mmol/L)	0.58(0.33,1.00)	0.04	0.29(0.15,0.54)	<0.01	0.30(0.15,0.61)	<0.01
LDL-C (mmol/L)	0.92(0.77,1,10)	0.39				
TC (mg/dl)	0.88(0.76,1.03)	0.12				
Cr (mg/dl)	1.00(0.99,1.01)	0.95			1.01(0.99,1.02)	0.14
RASi, n (%)	1.76(1.24,2.51)	<0.01	1.56(1.06,2.28)	0.02	1.30(0.85,1.99)	0.23
Hypertension, n (%)	1.51(1.06,2.14)	0.02			1.02(0.65,1.59)	0.94

CI, Confidence Interval; BMI, body mass index; SBP, systolic blood pressure; FBG, fasting blood-glucose; FCP, fasting C-peptide; HOMA-IR(CP), homeostatic model assessment of insulin resistance(C-peptide); ALT, alanine aminotransferase; AST, aspartate aminotransferase; HDL-C, high-density lipoprotein cholesterol; TC, total cholesterol; LDL-C, low-density lipoprotein cholesterol; RASi, renin-angiotensin system inhibitors.

Model 1: Non-adjusted.

Model 2: Adjusted for age, sex, BMI, Cr, hypertension.

Metabolic variables were the primary predictors for progression. In particular, the following factors remained significant in all the models as important protective variables: HOMA-IR (OR = 0.58, p < 0.01), Male (OR = 0.50, p = 0.01) and HDL-C (OR = 0.30, p < 0.01).

**Figure 2:**
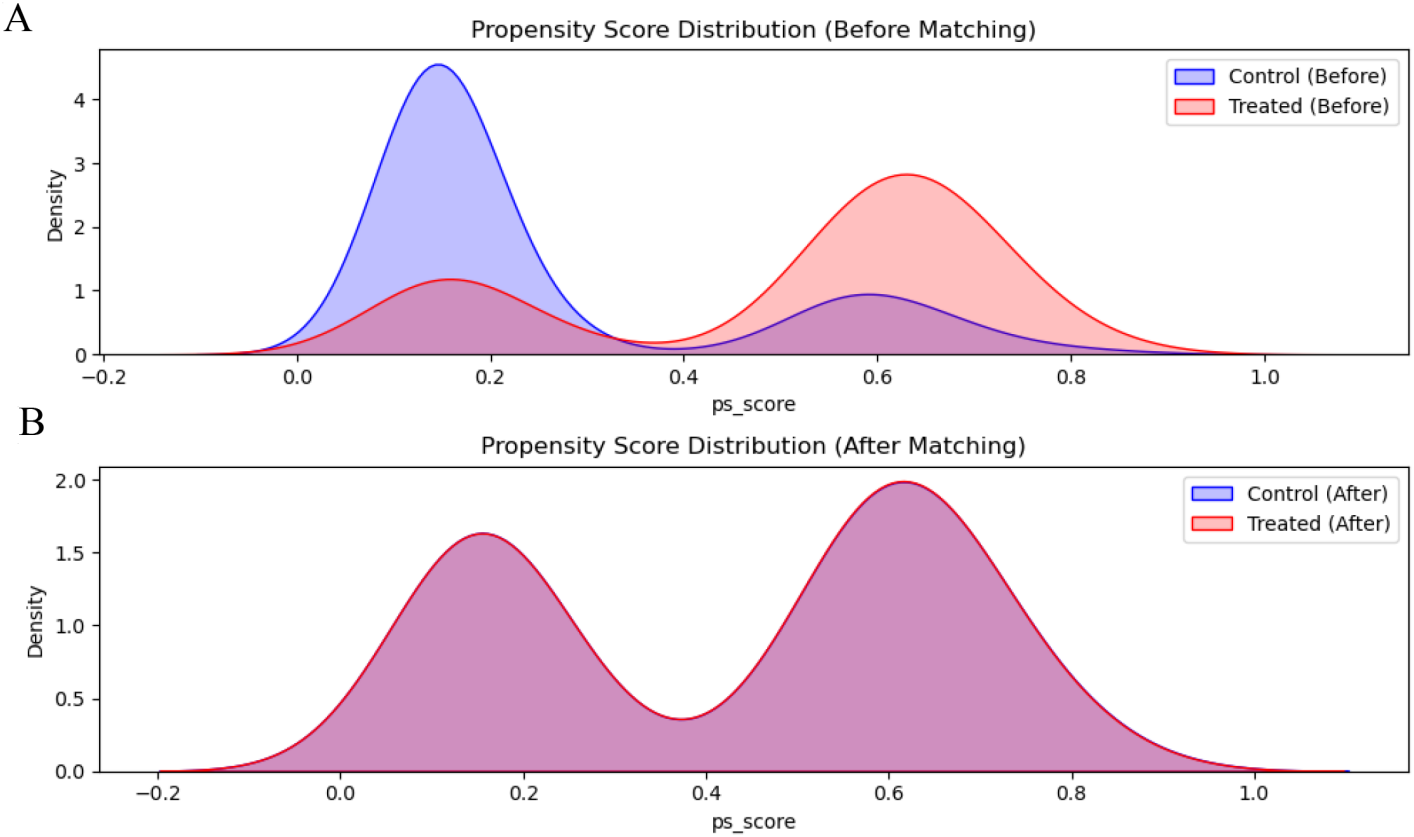
Distribution of propensity scores before and after propensity score matching (PSM). **(A)** Distribution of propensity scores in the original cohort (n=682) before matching. **(B)** Distribution of propensity scores in the matched cohort (n=246) after 1:1 matching. The red area represents the RASi group, and the blue area represents the non-RASi group. The substantial overlap in panel B indicates successful balancing of baseline covariates.

In the multivariate model, there were also small, significant, independent predictors for progression in BMI (OR = 1.06, p=0.03)However, given that RASi use is often confounded by indication bias (e.g., prescribed for severe hypertension), we performed a 1:1 PSM analysis. **Figure 2** illustrates the distribution of propensity scores, showing substantial overlap after matching. **Table 4** confirms that all baseline covariates, including blood pressure and eGFR, were well-balanced between the RASi and non- RASi groups (p>0.05).

**Table 4:** Baseline characteristics of patients with and without RASi treatment before and after propensity score matching.

Characteristics	Before matching (original cohort)			After matching (balanced cohort)		
	Non-RASi (n = 449)	RASi (n = 233)	P	Non-RASi (n = 123)	RASi (n = 123)	P
Sex			0.11			1.00
Male, n (%)	306 (68.2%)	144 (61.8%)		80 (65.0%)	80 (65.0%)	
Female, n (%)	143(31.8%)	89(38.2%)		43(35.0%)	43(35.0%)	
Age (years)	48.7 ± 11.8	54.2 ± 12.1	<0.01	51.7 ± 12.8	53.2 ± 12.5	0.368
SBP (mmHg)	127.8 ± 15.9	134.1 ± 16.9	<0.01	131.0 ± 16.4	132.5 ± 17.6	0.49
DBP (mmHg)	76.3 ± 9.7	77.3 ± 11.0	0.21	76.6 ± 10.0	77.6 ± 10.4	0.43
eGFR (mL/min/1.73m²)	107.6 ± 15.4	99.7 ± 17.9	<0.01	102.4 ± 16.8	102.0 ± 18.5	0.84
Hypertension, n (%)	98 (21.8%)	171 (73.4%)	<0.01	72 (58.5%)	71 (57.7%)	1.00
Family History of Diabetes, n (%)	240 (53.5%)	129 (55.4%)	0.69	61 (49.6%)	65 (52.8%)	0.70

SBP, systolic blood pressure; DBP, diastolic blood pressure; eGFR, estimated glomerular filtration rate;.

a Continuous variables were expressed as mean ± standard deviation for normally distributed data and as median (interquartile range, IQR) for skewed data. Categorical variables were reported as frequencies and percentages.

Finally, in the matched cohort, the association between RASi use and CKM progression was no longer statistically significant (OR = 1.56, 95% CI: 0.91–2.67; p = 0.156, **Table 5**). These results indicate that baseline comorbidities, rather than the medication itself, are the primary drivers of progression.

**Table 5:** Adjusted risk of CKM progression associated with RASi use in the original and propensity score-matched cohorts.

Cohort	Total (N)	Progression	Incidence	OR (95% CI)	P
Original Cohort (Before Matching)	682	172	25.2%		
Non-RASi	449	103	22.9%	Ref	
RASi	233	69	29.6%	1.41 (0.99-2.01)	0.05
Matched Cohort (After Matching)	246	69	28.0%		
Non-RASi	123	29	23.6%	Ref	
RASi	123	40	32.5%	1.56 (0.91-2.67)	0.156

CI, Confidence Interval; RASi, Angiotensin-Converting Enzyme Inhibitors/Angiotensin Receptor Blockers.

Furthermore, we performed a gender-based PSM analysis (150 matched pairs) to investigate the initially observed protective effect of male sex (OR = 0.50, p=0.01). After rigorously balancing baseline covariates, the risk of CKM progression showed no significant difference between males and females (OR = 0.868, 95% CI: 0.516–1.462; p=0.596, **Supplementary Table 2**). This confirms that the apparent gender-specific protection was also an artifact driven by baseline epidemiological variances.

## 4 Discussion

In this retrospective study of diabetic patients with COVID-19, we identified that while RASi use appeared as a risk factor in the preliminary multivariate regression analysis, PSM analysis revealed this to be a reflection of confounding by indication rather than progression risk caused by the drug itself. Moreover the seemingly paradoxical low HOMA-IR phenotype observed within the progression cohort is indicative of severe viral-induced β-cell secretory decompensation rather than enhanced insulin sensitivity while concurrently identifying elevated high-density lipoprotein cholesterol as a resilient protective determinant.

While preliminary multivariate logistic regression models implicated RASi usage as a potential determinant of CKM progression exhibiting an odds ratio of 1.56 this association is likely an artifact of confounding by indication inherent to retrospective designs wherein patients manifesting severe baseline comorbidities such as hypertension or albuminuria are preferentially prescribed these medications (18, 25). By using PSM to balance the baseline characteristics stringently, we showed that the significant association between the use of RASi and CKM progression disappeared, with a p-value of 0.156. This conclusion is supported by several recent large-scale studies, such as the one by Hamada et al. of 7,613 participants, which showed a similar lack of an independent association between the exposure to RAS inhibitors and mortality subsequent to strict PSM adjustment (26). Similarly, Kim et al. utilized Inverse Probability of Treatment Weighting (IPTW) to show that the risk was nullified after adjusting for baseline comorbidities (18). Large-scale randomized trials like REMAP-CAP further confirmed the neutral effect of RASi initiation in hospitalized COVID-19 patients (27).

Although RASi is no longer a statistically independent risk factor, the OR of 1.56 suggests a potential underlying trend, which may be linked to the dual role of ACE2. While RASi may theoretically facilitate viral entry by upregulating ACE2 receptors, ACE2 also exerts anti-inflammatory and anti-fibrotic effects that protect the endothelium (28). Zhang et al. even found that long-term RASi use was associated with reduced severity in elderly patients infected with the Omicron variant (29). Therefore, in our cohort, RASi use serves more likely as a “marker of severity” for high-risk patients rather than a direct pathogenic driver.

The homeostasis model assessment (HOMA), a cornerstone metric for evaluating insulin sensitivity and β-cell function, and its derivative parameter HOMA-IR, are widely utilized in clinical assessments of insulin resistance (30). In our study, the CKM progression group exhibited significantly lower HOMA-IR scores (OR = 0.34, 95% CI: 0.20–0.67; p < 0.01). However, this should not be misinterpreted as improved insulin sensitivity. When considered alongside the significantly reduced fasting C-peptide (p < 0.01) and elevated FBG (p < 0.01) observed in the progression group, this profile reflects a state of severe pancreatic β-cell secretory dysfunction.

We start with the hypothesis that SARS-CoV-2 infection causes a substantial aggravation of insulin resistance in diabetic patients through multiple mechanisms. This is because He et al. have shown that viral-induced reduction in REST levels disrupts glucose and lipid metabolism regulators such as myeloperoxidase, apelin, and myostatin, thereby directly contributing to hyperglycemic events (31). Alongside, there is activation of the Integrated Stress Response (ISR) triggered by viral RNA fragments that activate double-stranded RNA-dependent protein kinase, ultimately resulting in serine phosphorylation of insulin receptor substrate(IRS), thereby reducing insulin signaling (32). This further worsens because of the cytokine storm that further activates serine/threonine kinases associated with ISR. When compounded with the chronic subclinical inflammation pervasive in diabetic patients—who are predisposed to elevated TNF-α and IL-6—these factors culminate in a state of systemic inflammatory overactivation, thereby propelling patients into severe insulin resistance (32, 33).

Secondly, new findings also suggest that SARS-CoV-2 can directly infect pancreatic β-cells through ACE2 receptors, leading to apoptosis or dedifferentiation (34, 35). In this study, the increased HOMA-IR value in the non-progression group represents the potential of the β-cells to compensate for severe insulin resistance by hypersecretion. In contrast, in the CKM progression group, the cumulative effects of several pathologic injuries have depleted this potential: virus-triggered β-cell dedifferentiation reduces the mass of functional cells, and the cytokine storm strongly promotes severe endoplasmic reticulum (ER) stress (36). Notably, the above-mentioned activation of the Integrated Stress Response (ISR) system also contributes to increased ER stress in β-cells, ultimately leading to functional paralysis (32). Therefore, these patients have entered a stage of “Islet Functional Failure” (Secretory Dysfunction) from a stage of “Insulin Resistance” (Metabolic Stress) (37).

Thus, in this specific pathophysiologic state, the HOMA-IR index transcends its classical role in merely assessing insulin resistance. In particular, when its levels are elevated, there is a phase of compensation, whereas when there is a paradoxical decrease along with decreased C-peptide levels, there is a transition into a phase of decompensation, marking the initiation of injury in multiple organs.

The inverse association between atherosclerosis pathogenesis and high-density lipoprotein cholesterol (HDL-C) is based on its pivotal role in reverse cholesterol transport, in addition to its antioxidant effects, hence defining HDL-C as an independent risk factor in a manner different from low-density lipoprotein cholesterol (LDL-C) (38). The shielding paradigm is applicable to viral susceptibility, which was shown by Vignesh et al. in a study conducted in the Arkansas Clinical Data Repository to be associated with a higher susceptibility to SARS-CoV-2 infection in persons with a low pre-infection concentration of HDL-C (39). Such findings are further corroborated by meta-analytical evidence demonstrating that admission HDL-C levels are markedly suppressed in critically ill phenotypes and non-survivors relative to non-critical cases and survivors respectively [pooled mean difference: -4.4 mg/dL and -2.5 mg/dL] thereby reinforcing the prognostic significance of lipid depletion in COVID-19 progression (40). These findings align with our observation that elevated HDL-C significantly reduced CKM syndrome progression risk (OR = 0.29).

Diabetic patients are prone to complications such as immune imbalance, excessive inflammatory response, and multi-organ functional damage after infection with new crowns, leading to more complex conditions and a significantly higher probability of critical illness (41, 42), whereas HDL-C reduces the release of inflammatory cytokines, such as TNF-α and IL-1B, by inhibiting the activation of nuclear factor kB (NF-kB) and down-regulating the expression of endothelial adhesion molecules (E-selectin, VCAM-1, etc.) expression, thereby alleviating the COVID-19-associated “inflammatory storm” (43, 44). In addition, diabetic patients had a significantly increased risk of cardiovascular events after infection with COVID-19 (10),Oxidized low-density lipoprotein (oxLDL) can induce vascular endothelial cell dysfunction and endothelial cell damage itself, and HDL-C can bind and take away oxLDL and reduce its deposition in the vessel wall (44).Through RCT, HDL-C facilitates hepatic clearance of macrophage-derived and plaque-embedded cholesterol, thereby suppressing foam cell generation and attenuating atherogenesis by lowering systemic and vascular cholesterol burdens (45, 46). This effect may exert additional protection by inhibiting virus-induced metabolic disturbances in diabetic patients with COVID-19.

Notably, the chronic low-grade inflammatory state in diabetic patients may lead to abnormal HDL function (e.g., reduced activity of the antioxidant enzyme PON1), resulting in the paradoxical phenomenon of “normal HDL-C levels but defective function,” which may partly explain why some patients develop CKM syndrome despite normal HDL-C levels progression (47).

Furthermore, an analysis of our baseline cohort (**Table 1**) reveals a notable male predominance in advanced CKM stages (Stages 2, 3, and 4, comprising 64.6%, 79.6%, and 67.1% of patients, respectively). This demographic skew aligns with established cardiovascular epidemiology. Biologically, pre-menopausal women benefit from the robust atheroprotective and metabolic-regulating effects of endogenous estrogen, which typically delays the onset of cardiovascular complications compared to men (48, 49). Additionally, behavioral risk factors significantly contribute to this disparity; in our cohort, smoking (268/450 vs. 10/232) and alcohol consumption (310/450 vs. 50/232) were predominantly observed in the male population (50, 51). Consequently, male patients entered the COVID-19 infection period with a substantially heavier pre-existing cardiorenal and metabolic burden.

In our initial multivariate logistic regression, male sex emerged as a significant independent protective factor against CKM progression (OR = 0.50, p=0.01). To elucidate whether this was a genuine biological advantage or an artifact of baseline confounding, we performed a gender-based Propensity Score Matching (PSM) analysis. After rigorously matching 150 male-female pairs across key baseline covariates (including age, blood pressure, renal function, and medication history), the post-infection CKM progression rates between females (26.7%) and males (24.0%) showed no significant statistical difference (OR = 0.868, 95% CI: 0.516–1.462; p=0.596). This crucial finding indicates that the apparent protective effect of male sex was driven by baseline epidemiological variances. In essence, because men had already accumulated significant CKM damage prior to infection, their relative rate of new progression appeared blunted compared to women, who experienced a noticeable “catch-up” deterioration post-infection (49). However, once the baseline metabolic and cardiovascular severity is standardized, the biological vulnerability to SARS-CoV-2-induced CKM progression is fundamentally comparable between both sexes.

The present study is distinguished by several pioneering contributions notably being the inaugural investigation to systematically dissect the determinants of CKM syndrome advancement within diabetic COVID-19 cohorts using the integrated CKM framework while simultaneously adjudicating the RASi safety debate through PSM which effectively minimized indication bias to clarify these medications as markers of baseline disease severity rather than independent pathogenic drivers. Moreover, we have offered mechanistic into the paradoxical HOMA-IR phenotype because we have identified viral-induced β-cell secretory decompensation as the chief mechanism for disease progression, in addition to insulin resistance. Simultaneously, we have increased our methodological sophistication with AHA PREVENT risk stratification for 10-year risk.

However, the study has several limitations, including the relatively small number of participants, which could pose selection bias, thus not being generally representative of the larger population. In addition, although the study has employed the use of propensity score matching, the study’s retrospective nature does not allow for the determination of causality, with the possibility of confounders such as individual cytokines and viral loads not being controlled for. Lastly, the study does not involve the long-term follow-up of the changes in the levels of CKM and the functional recovery of the pancreatic β-cells.

## 5 Conclusion

In summary, our study suggests that the progression of CKM syndrome in diabetic patients following COVID-19 is likely driven by a complex interplay of baseline comorbidity burden and viral-induced metabolic decompensation. While initial analyses associated RASi use with adverse outcomes, our propensity-matched results indicate that this relationship is largely confounded by indication. Therefore, RASi use should be interpreted with caution—potentially serving as a marker of underlying disease severity rather than a direct driver of progression. Mechanistically, we propose that SARS-CoV-2 infection may accelerate the transition from “insulin resistance” to “β-cell secretory dysfunction” (manifested as acute insulinopenia and paradoxical low HOMA-IR), a process likely fueled by direct viral toxicity and inflammatory stress. Conversely, HDL-C exhibits a robust protective role. Clinical implications of these findings suggest that routine discontinuation of RASi may not be warranted, but careful monitoring of renal and hemodynamic status is essential in these high-risk patients. Future management strategies should prioritize the preservation of islet function and the optimization of lipid profiles to mitigate the risk of multi-organ damage in the post-COVID era.

## Data Availability

The raw data supporting the conclusions of this article will be made available by the authors, without undue reservation.
