# Supplementary material for: COVID-19 associated CKM syndrome progression in diabetic patients is linked to pancreatic beta cell dysfunction, rather than RASi use: a retrospective cohort study
Source: Front Endocrinol (Lausanne). 2026 Mar 9;17:1790281. doi: 10.3389/fendo.2026.1790281 (PMC13007547; doi:10.3389/fendo.2026.1790281)
Supplement: Supplementary file 1 [file DataSheet1.docx]

Supplementary Material

# Supplementary Figures and Tables

Table S1. Multivariable Logistic Regression Model for Estimation of Propensity Scores for RASi Prescription

| Characteristics | Coefficient(β) | Odds Ratio (95% CI) | P |
| --- | --- | --- | --- |
| Sex (Male) | -0.072 | 0.93 (0.62 – 1.40) | 0.73 |
| Age (years) | 0.001 | 1.00 (0.98 – 1.02) | 0.936 |
| SBP (mmHg) | 0.017 | 1.02 (1.00 – 1.03) | 0.030 |
| DBP (mmHg) | -0.002 | 1.00 (0.97 – 1.02) | 0.895 |
| eGFR (mL/min/1.73m²) | -0.011 | 0.99 (0.97 – 1.00) | 0.116 |
| Hypertension, n (%) | 2.119 | 8.32 (5.64 – 12.29) | <0.01 |
| Family History of Diabetes, n (%) | 0.128 | 1.14 (0.78 – 1.66) | 0.508 |

Abbreviations: CI, Confidence Interval; SBP, systolic blood pressure; DBP, diastolic blood pressure; eGFR, estimated glomerular filtration rate; RASi, renin-angiotensin system inhibitors.

Figure S1. Distribution of propensity scores by gender before and after propensity score matching (PSM).


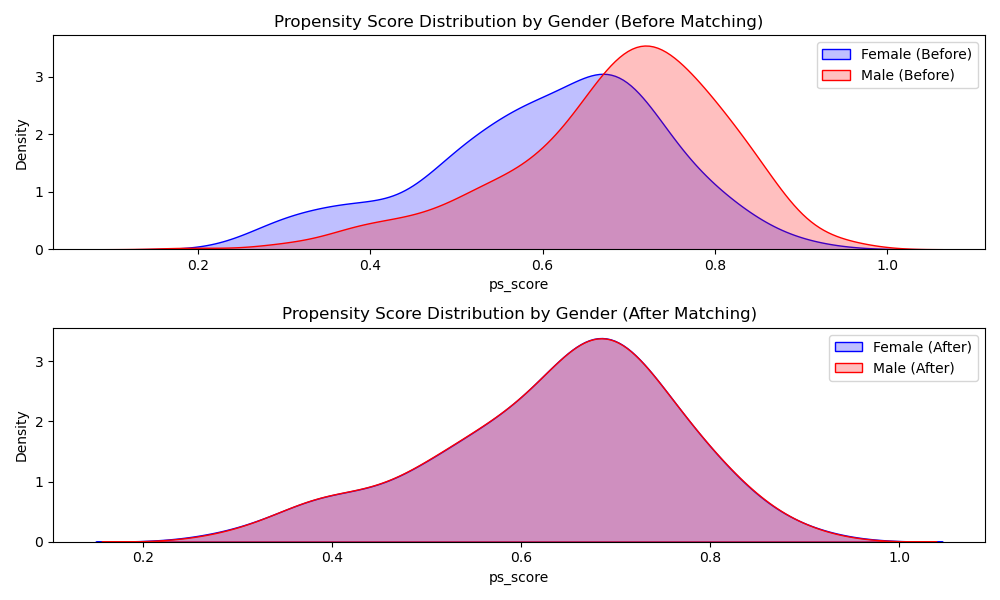


(A) Distribution of propensity scores in the original cohort (n=682) before matching. (B) Distribution of propensity scores in the matched cohort (n=300) after 1:1 matching. The red area represents the Male group, and the blue area represents the Female group. The substantial overlap in panel B indicates successful balancing of baseline covariates.

Table S2. Incidence and risk of CKM progression between male and female patients in the propensity score-matched cohort

| Sex | Total(n) | CKM Progression, n (%) | Odds Ratio (95% CI) | P |
| --- | --- | --- | --- | --- |
| Female | 150 | 40(26.7%) | Ref |  |
| Male | 150 | 36(24.0%) | 0.868 (0.516–1.462) | 0.596 |

Abbreviations: CKM, Cardiovascular-Kidney-Metabolic; CI, Confidence Interval; Ref., Reference category.

The cohort was 1:1 matched by Propensity Score Matching (PSM) for baseline covariates including age, systolic and diastolic blood pressure, estimated glomerular filtration rate (eGFR), hypertension history, family history of diabetes, and RASi usage, effectively eliminating baseline demographic and clinical disparities between genders.
